# Clinical Outcomes of Primary Posterior Continuous Curvilinear Capsulorhexis in Postvitrectomy Cataract Eyes

**DOI:** 10.1155/2020/6287274

**Published:** 2020-07-30

**Authors:** Mengting Yu, Duan Yan, Wenjie Wu, Yingbin Wang, Xinna Wu

**Affiliations:** ^1^Ophthalmology Department, Provincial Clinical Medical College of Fujian Medical University, Fuzhou 350001, China; ^2^Ophthalmology Department, Fujian Provincial Hospital, Fuzhou 350001, China

## Abstract

**Purpose:**

To evaluate the safety and outcomes of primary posterior continuous curvilinear capsulorhexis (PPCCC) combined with phacoemulsification in postvitrectomy eyes.

**Design:**

Retrospective case series.

**Methods:**

Twenty-one postvitrectomy eyes of 21 patients with cataract between April 2017 and December 2019 were enrolled. PPCCC through the cornea incision was performed before in-the-bag intraocular lens implantation. All patients were followed up for at least 3 months postoperatively. The outcome measures were corrected distance visual acuity (CDVA), intraocular pressure (IOP), corneal endothelium cell counts (CECC), central macular thickness (CMT), the occurrence of intraoperative or postoperative complications, and the incidence of posterior capsular opacification (PCO).

**Results:**

The mean age was 56.14 ± 9.76 years (ranging from 31 to 68). The mean Snellen CDVA was 20/400 preoperatively and improved to 20/67 postoperatively (*P* < 0.001). No significant differences were found between IOP (*P* = 0.96) and CMT (*P* = 0.42) preoperatively and postoperatively. The mean CECC was 2571.8 ± 319.3 cells/mm^2^ preoperatively and 2498.2 ± 346.3 cells/mm^2^ postoperatively (*P* < 0.05). Lens epithelium cells proliferation was confined to the peripheral capsular bag during a mean follow-up of 12.9 ± 10.5 months (ranging from 3 to 28 months). Intraoperative posterior capsule extension occurred in 1 eye (4%), although it did not affect the patient's vision. No serious complications, including retinal detachment or endophthalmitis, were detected in any of the 21 cases.

**Conclusion:**

PPCCC through cornea incision combined with phacoemulsification may be a safe and practical alternative to prevent PCO in postvitrectomy eyes with cataract.

## 1. Introduction

In recent years, improvements in surgical technique and advances in instruments facilitate the widespread use of vitrectomy in the management of posterior segment disorders. Vitrectomy has been shown to bring about accelerated formation and progression of cataract [1, 2]. It is reported that 80–100% of phakic eyes require cataract surgery within 2 years following vitrectomy [2, 3], which has made postvitrectomy eyes a significant proportion of cataract eyes and has aroused more concerns of ophthalmologists for this growing population. What's more, the formation of posterior capsular opacification (PCO) is more rapid and extends in patients undergoing vitrectomy [4]. It is confusing for these patients confronted with vision loss again in the early postoperative period, which lowers the patients' satisfaction significantly. Besides, the opacified region also troubles doctors by hindering adequate visualization of the retina. The standard treatment to solve this problem is creating a posterior capsular opening via neodymium-doped yttrium aluminum garnet (Nd : YAG) laser, whereas Nd : YAG is not accessible in some developing and underdeveloped areas in China, besides some less-developed regions in the world. Those who develop PCO after vitrectomy have to be referred to the previous tertiary ophthalmic center again to receive laser posterior capsulotomy, which poses further time and cost burden for these patients virtually.

One alternative of the Nd : YAG laser is to perform a primary posterior capsular continuous curvilinear capsulorhexis (PPCCC) at the time of cataract surgery, which may avert vision deterioration in the postvitrectomy eye. Nowadays, PPCCC is widely used in pediatric cataract surgery in the hope of preventing PCO, but not routinely adopted in adult cataract surgery, attributing to concerns regarding associated complications, including vitreous interface damage, vitreous prolapse, resulting cystoid macular edema (CME), and even retinal detachment (RD) [5, 6]. Though previous studies have demonstrated its safety and efficacy in age-related cataract [7–9], most inexperienced surgeons may still hesitate to this technical demanding manipulation considering the potential complications mentioned above.

However, we speculate that compared with nonvitrectomy eyes, PPCCC may carry lower risks in postvitrectomy eyes on account of absent vitreous body. To date, few studies have evaluated the use of PPCCC at the time of cataract surgery in postvitrectomy eyes of adults. From 2017 to 2019, we performed PPCCC with capsular forceps through the corneal incision during cataract surgery in postvitrectomy eyes. The safety, feasibility, efficacy, and clinical outcomes of PPCCC combined with phacoemulsification in postvitrectomy eyes are shown as follows.

## 2. Materials and Methods

### 2.1. Patients

This was a retrospective study conducted at the ophthalmology department, Fujian Provincial Hospital, between April 2017 and December 2019. Postvitrectomy eyes were enrolled consecutively. Exclusion criteria included patients with uveitis, trauma, pseudoexfoliation syndrome, and connective tissue diseases. The present research adhered to the tenets of the Declaration of Helsinki and was approved by the Institution Review Board of Fujian Provincial Hospital, Fujian Medical University. The informed consent was obtained from all the patients.

All patients went through a comprehensive ophthalmologic examination preoperatively and at the last follow-up, including Snellen corrected distance visual acuity (CDVA), IOP measure, corneal endothelial cell count (CECC), slit-lamp examination, fundus examination, central macular thickness (CMT) measurement using spectral domain optical coherence tomography (OCT) (Spectralis, Heidelberg Engineering GmbH), and anterior segment retroillumination photograph (SL-D7, TOPCON). The rate of complications and the incidence of PCO were recorded.

### 2.2. Surgical Technique

**Figure 1:**
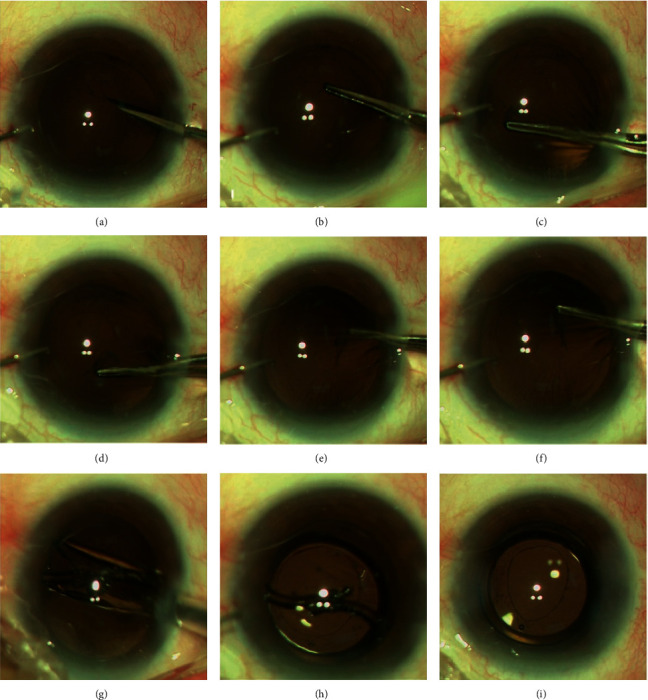
Illustration of posterior capsulotomy using capsular forceps from the cornea incision. (a) The central part of the posterior capsule was punctured with a 27-gauge needle. (b–f) Forceps were introduced through the main incision to grasp the peripheral edge of the fissure to create a well-centered and round PPCCC with a diameter of 4-5 mm in size. (g–i) A foldable acrylic one-piece IOL was injected into the anterior chamber using a cartage. We paid attention to keep the main body of the IOL above the anterior capsule in case that the IOL tilt or dislocate from the posterior opening. After that, a spatula was used to adjust and rotate the IOL into the capsular bag.

Surgery was performed by an experienced surgeon (WJ. W). Preoperatively, compound tropicamide eye drops were used for pupil dilation and proparacaine eye drops were applied for topical anesthesia. The major procedures of phacoemulsification were performed as conventional surgery. The anterior continuous curvilinear capsulorhexis (ACCC) with a diameter of 5.5 mm, nucleus removal, cortical aspiration, and posterior capsule polishing were performed. The capsule bag was filled with an ophthalmic viscosurgical device (OVD) (sodium hyaluronate 15 mg/ml, Shanghai Qisheng Biological Preparation Co., Ltd.). The central part of the posterior capsule was punctured with a 27-gauge needle from the center to the peripheral to create a 2 mm crescent-shaped fissure, and the capsule bag was refilled with OVD. Forceps were introduced through the main incision to grasp the peripheral edge of the fissure to create a flap. The edge of the flap was grasped and regrasped every quarter circle, enabling a well-centered and round PPCCC with a diameter of 4-5 mm in size (shown in Figure 1). Care should be taken to lift towards the center and upward along the tangent direction during PPCCC procedure. A foldable acrylic one-piece IOL (Tecnis ZCB00, J&J Vision Inc.) with a 6.0 mm round optic was injected into the anterior chamber using a cartage. We paid attention to keep the main body (optic) of the IOL above the anterior capsule in case that the IOL tilt or dislocate from the posterior capsulotomy. After that, a spatula was used to adjust and rotate the IOL into the capsular bag. The residual OVD was aspirated and the surgical wounds were watertight.

13 out of 21 eyes coexisted with the residual silicone oil droplets in the vitreous cavity.

After PPCCC and before the IOL implantation, the silicone oil droplets were guided and removed by a 21-gauge infusion-aspiration syringe from the main incision. Then, the IOL was implanted and the OVD was aspirated from the anterior and posterior chambers. At last, the surgical wounds were watertight.

All patients received topical antibiotics, corticosteroids, and nonsteroidal anti-inflammatory eye drops for 4 weeks postoperatively.

### 2.3. Statistical Analysis

Snellen visual acuity was converted to logMAR units for comparisons. We used a logMAR VA of 2 and 3 to represent counting fingers and hand move vision [10]. Corrected distance visual acuity, corneal endothelial cell density, intraocular pressure, and central macular thickness were compared between pre- and postprocedural levels. The means and standard deviations (SD) of the quantitative variables were calculated. The paired *t*-test was used to detect the differences of quantitative variables when data obeyed normal distribution; otherwise, the Wilcoxon matched-pairs signed ranks sum test was used. The differences were considered statistically significant if the *P* value was less than 0.05. All calculations were performed using SPSS software (version 24, SPSS, Inc.).

## 3. Results

**Figure 2:**
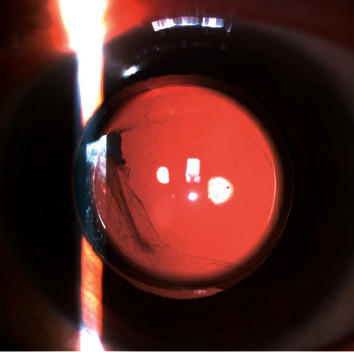
Slit-lamp photograph (retroillumination) of the PPCCC at 3 months postoperatively. Though capsular irregular tear occurred intraoperatively, PPCCC could be completed assisted by capsule scissors.

**Figure 3:**
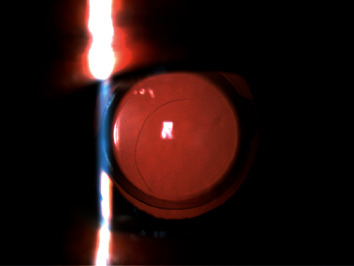
Slit-lamp photograph (retroillumination) of the PPCCC at 3 months postoperatively.

**Figure 4:**
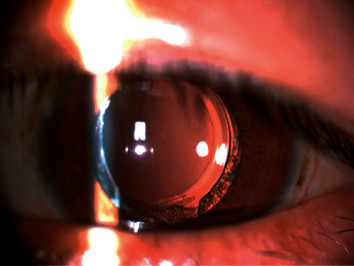
Slit-lamp photograph (retroillumination) of the PPCCC at 26 months postoperatively; Elschnig-pearl-type PCO was visible in the remaining periphery capsule.

**Figure 5:**
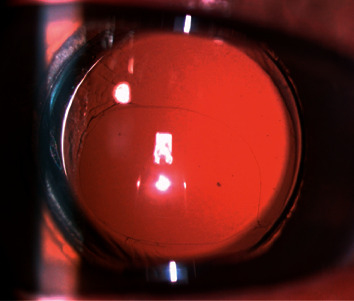
Slit-lamp photograph (retroillumination) of the PPCCC at 3 months postoperatively; fibrosis-type PCO was visible in the remaining periphery capsule.

PPCCC combined with phacoemulsification was performed in 21 postvitrectomy eyes of 21 adult patients. The demographic characteristics are shown in Table 1. The mean age of the 9 women and 12 men was 56.14 ± 9.76 years (ranging from 31 to 68) when the surgery was performed. The previous reasons requiring vitrectomy were rhegmatogenous retinal detachment in 15 (71.4%) eyes, branch retinal vein occlusion in 1 (4.7%) eye, and proliferative diabetic retinopathy in 5 (23.8%) eyes. No severe intraoperative and postoperative complications, including IOL dislocation or subluxation, retinal detachment, or endophthalmitis, were detected in any of the 21 cases. Capsular irregular tear occurred in 1 (4.7%) eye, but PPCCC could be completed assisted by capsule scissors (shown in Figure 2). 4 (19%) eyes with posterior capsular fibrotic plaque were observed intraoperatively. Slightly decentred opening was observed in 2 (9.6%) patients. Appropriate IOL centration and in-the-bag fixation was achieved in the rest of the eyes. During the follow-up time, the visual axis was clear in all eyes (shown in Figure 3), and mild to severe lens epithelium cell growth was visible in the remaining periphery capsule in 3 eyes (shown in Figures 4 and 5), and Nd : YAG laser posterior capsulotomy was not required during the follow-up.

The mean CDVA. ECED, IOP, and CMT preoperatively and at the last visit are shown in Table 2. Visual acuity improved in all cases. The mean CECD decreased slightly between preoperative and last visit, and this small difference was statistically significant. The mean IOP and mean CMT did not change significantly. At the last visit, macular edema was observed in 3 of the 21 patients. Two of those had a diabetic macular edema preoperatively and improved after anti-VEGF treatment. New onset macular edema was found in 1 (4%) eye 6 months postoperatively and ameliorated after topical administration of nonsteroidal anti-inflammatory drugs. The mean follow-up time was 12.9 ± 10.5 months (ranging from 3 to 28 months).

## 4. Discussion

The most common long-term complication of cataract surgery is posterior capsule opacification. Although PCO has been reduced by modified IOL designs and improved surgical techniques, it cannot be completely eliminated. Furthermore, in previous vitrectomized eyes, PCO proceeds faster compared to the nonvitrectomy eyes [11–13], depriving the patients of their best potential vision even in the early postoperative period. This discrepancy was mainly caused by a loss of vitreous compression. Theoretically, the vitreous cavity is filled mainly with liquid after PPV, which exerts less pressure on the posterior compared to positive vitreous body, leaving a larger space between the posterior capsule and IOL posterior surface. Nishi et al. [14] suggested that capsular bend formation is the mainstay of edge effect in square edge IOL. In vitreous-removed eyes, the posterior capsule exerts less force on the optic edge according to the loss of compression theory [13]. This phenomenon prohibits the rapid angle formation in the capsule-optic apparatus, which may make LEC migration and proliferation easier. Early-onset PCO in postvitrectomy eyes not only has a severe impact on the patients' quality of life but also influences the scrutinized fundus examination by ophthalmic specialists.

Nd : YAG laser capsulotomy, commonly used in clinically significant PCO eyes, carries risks including a rise in IOP, uveitis, damage or dislocation of the IOL, vitreous prolapse, vitreous floaters, CME, and even RD [15–17]. Moreover, there are still concerns about the cost-effectiveness of Nd : YAG because the treatment might pose a considerable burden on patients and the medical care system. Nd : YAG is not universal in developing and underdeveloped areas in China, so the patients with early-onset PCO will turn to the previous tertiary ophthalmic center again for laser posterior capsulotomy, which may bring about undue anxiety and increase time, travel, and health-care expense.

PPCCC was first introduced in the presence of residual posterior capsular opacity that could not be polished completely, or in the prevention of extension of an inadvertent posterior capsule tear intraoperatively. It is applied gradually to clear and intact postcapsule, especially in pediatric cataract surgery in order to prevent vision deterioration caused by PCO. In addition, compared with Nd : YAG laser capsulotomy, it has lower risks of the laser-related complications and no capsular or cellular debris left in the eye. Furthermore, Kim and Kim [8] demonstrated PPCCC is useful to stabilize the IOL and minimize postoperative refractive changes. However, this approach is not performed routinely in age-related cataract surgery on account of the potential complications in regard to vitreous interface disruption and higher technique requirement. Nevertheless, there are some specific features in the postvitrectomy eyes compared to the normal ones. In the normal eyes, the fibrillary gel structure of vitreous might provide a mechanical support for the lens-capsule complex during phacoemulsification procedure. On the contrary, less support for the lens-capsule complex was observed when vitreous gel had been removed and replaced with saline solution. It was possible that in supine position, saline solution is more influenced by gravity than normal vitreous gel structure, which may enable a larger Berger space (between the posterior capsule and the anterior hyaloid membrane). The pressure near the posterior capsule might decrease compared to the normal cataract eyes, which reduces the possibility and severity of the vitreous body herniation. Based on the aforementioned states, we hypothesize the lower risk of capsule-related complications is associated with the reduced pressure of vitreous cavity and less amount of vitreous material. The primary PPCCC was completed in all cases among our study. Although there was an irregular capsular tear during the procedure in 1 (4.8%) eye, PPCCC was still accomplished assisted by the capsular scissors. A new flap was created at an appropriate position (2 mm away from the center) along the rim of tear, and then PPCCC was continued successfully. It demonstrated that PPCCC could still be performed with caution in circumstance of irregular capsular tear (Figure 5). However, it is recommended to back up a three-piece IOL where a primary PCCC is planned, and in the event where it is not possible to place the IOL in the bag, a three-piece IOL with haptics in the sulcus and optic capture of the optic through the anterior capsulorhexis should be done. 4 (19%) eyes with posterior capsular fibrotic plaque were observed intraoperatively. It is recommended that careful consideration should be taken in eyes with posterior capsular fibrotic plaque: the central plaque can be removed simultaneously when PPCCC is performed; the peripheral plaque of the capsule should be circumvented in case of uncontrolled extension (Figure 5). Slightly decentered opening was observed in 2 (9.6%) patients possibly attributed to relatively lax capsule intraoperatively, while it did not affect the vision and IOL position (Figure 4). A well-centered and regular capsular opening was observed in the rest of the patients (Figure 2). Though fibrosis distributed on the remaining capsule was visible in 16 (76.2%) eyes, the opening of PPCCC kept transparent, which benefited both patients and doctors. Therefore, Nd : YAG laser posterior capsulotomy was not required during the follow-up. No intraocular and postoperative complications such as IOL dislocation into the vitreous cavity, RD, and endophthalmitis were encountered. In our study, no hypotony or elevated intraocular pressure occurred intraoperatively and postoperatively. One effective way to maintain IOP intraoperatively is to complement OVD, especially during the capsulorhexis procedure. It was recommended that OVD should be injected on the surface of the remaining posterior capsule rather than the rim of capsulorhexis to prevent iatrogenic posterior capsular tear and OVD left in the vitreous cavity.

It has been argued that creating a nonanatomical pathway between the anterior segment and posterior segment would induce complications including higher possibility of CME, RD, and endophthalmitis. De Groot et al. [18] found that a surgical posterior capsule curvilinear opening did not disrupt the aqueous-vitreous barrier 1 year after cataract surgery. Our study demonstrated no retinal detachment and endophthalmitis occurred. Diabetic macular edema was found in 2 (8%) eyes preoperatively. After anti-VEGF therapy, the macular thickness in both eyes reduced (from 402 um to 323 um and 706 um to 395 um, respectively) at the last visit. New onset macular edema was found in 1 (4%) eye 6 months postoperatively, which presented only increased CRT (from 398 um to 343 um) rather than typical cystoid changes as Irvine-Gass Syndrome. We supposed postoperative chronic inflammation was the underlying mechanism of the edema, which was ameliorated after topical administration of nonsteroidal anti-inflammatory drugs 4 times daily for a month.

A slight decrease in CECD postoperatively was observed in our study, which was in accordance with previous researches [19, 20]. Previous studies demonstrated nuclear sclerotic cataract progresses within 2 years after vitrectomy [1]. 21 eyes in our study were all sclerotic nuclear cataract, which requires more phaco energy, predisposing to the decreased CECD. Two patients had a loss of 323 and 426 cells/mm, respectively, because of relatively sclerotic nuclear (level 4, LOCS III).

The recommended size of the PPCCC varied from 3 to 5 mm in different studies [6, 8, 21]. In terms of the optimum size of the posterior opening, optical and mechanical factors should be taken into account. On one hand, optical factors require a large transparent zone, which supplies ophthalmologists with a wider view of retinal inspection. These postvitrectomy individuals who are in need of frequent, detailed, and widefield fundus examination may benefit a lot from the clear and size-substantial optical pathway. On the other hand, mechanical considerations favor a small posterior capsulotomy to afford adequate support for the IOL in-the-bag stabilization. On the basis of our experience, the optimum size of PPCCC is 4-5 mm approximately which satisfied both optical and mechanical factors. Furthermore, in our study, 13 out of 21 eyes coexisted with residual silicone oil droplets in the vitreous cavity. These patients presented with significant visual disturbance caused by the residual silicone oil droplet. Sakimoto et al. [22] reported the droplet could be aspirated with the 25-gauge vitreous cutter by two-port pars plana vitrectomy using 25-gauge instruments. We managed to use a 21-gauge infusion-aspiration syringe introduced from the PPCCC to guided and removed the residual silicone oil droplets from the main incision, which was proved to be safe, convenient, economical, and independence of advanced instruments.

The limitations of the study include its retrospective nature, relatively small sample size, short follow-up time, and steeper learning curve of this procedure. This approach is not an extra technical demanding procedure for those well-trained cataract surgeons whose operation volume is over 1000 cases and experienced in CCC techniques. Otherwise, larger samples and multicentre studies may still be warranted owing to the growing population of postvitrectomy cataract cases. Since the Nd : YAG laser is not available in primary hospitals in developing and underdeveloped areas, the practical approach is more substantially significant for the old, the disabled, and those living far away from the tertiary hospitals. These patients can avoid blurred vision caused by PCO and repeated doctor's appointments, which reduces the transport and medical expense.

## 5. Conclusions

In conclusion, PPCCC through the cornea incision during cataract surgery may be a safe, practical, and cost-effective alternative in the postvitrectomy eyes.

## Figures and Tables

**Table 1:** Demographic characteristics of the patients with postvitrectomy cataract eyes undergoing PPCCC.

Parameter	
Age (year)	56.14 ± 9.76
Eye (OD/OS)	13/8
Gender (M/F)	12/9
Reasons for PPV	
RRD (%)	71.4
BRVO (%)	4.7
PDR (%)	23.8
Follow-up time (months)	12.9 ± 10.5

PPV = par plana vitrectomy; RRD = rhegmatogenous retinal detachment; PDR = proliferative diabetic retinopathy; BRVO = branch retinal vein occlusion.

**Table 2:** Preoperative and postoperative IOP and CMT.

Parameter		Pre	Post	*P* value
CDVA (logMAR)				
Mean ± SD		1.57 ± 0.55	0.64 ± 0.32	*P* < 0.001†
Range		0.7–3	0.1–1.22	
CECD (cells/mm^2^)				
Mean ± SD		2571.8 ± 319.3	2498.2 ± 346.3	*P* < 0.001‡
Range		1938.2–3384.9	1787.2–3277.2	
IOP (mmHg)				
Mean ± SD		15.52 ± 2.06	15.57 ± 2.00	*P*=0.96†
Range		12–22	12–21	
CMT (*µ*m)				
Mean ± SD		249.71 ± 43.49	265.29 ± 64.53	*P*=0.42†
Range		213–316	202–398	

CDVA = corrected distance visual acuity; CECC = corneal endothelial cell count; Pre = preoperatively; Post = postoperatively; IOP = intraocular pressure; CMT = central macular thickness. †Wilcoxon matched-pairs signed ranks sum test between the eyes preoperatively and at the last follow-up. ‡Paired *t*-test between the eyes preoperatively and at the last follow-up.

## Data Availability

The dataset analyzed during the current study may be available from the corresponding author upon reasonable request.
